# Integrated Design of Electrically Configurable Ferroelectric and Redox‐Based Memristors for Hardware‐Implemented Reservoir Computing

**DOI:** 10.1002/advs.202505688

**Published:** 2025-06-10

**Authors:** Jung‐Kyu Lee, Yongjin Park, Euncho Seo, Jong‐Ho Lee, Sungjoon Kim, Sungjun Kim

**Affiliations:** ^1^ Department of Semiconductor Engineering Gyeongsang National University Jinju Gyeongnam 52828 Republic of Korea; ^2^ Department of Electrical and Computer Engineering and Inter‐university Semiconductor Research Center Seoul National University Seoul 08826 Republic of Korea; ^3^ Department of AI Semiconductor Engineering Korea University Sejong 30019 Republic of Korea; ^4^ Division of Electronics and Electrical Engineering Dongguk University Seoul 04620 South Korea

**Keywords:** ferroelectric, hafnia, memristor, multifunction, reservoir computing

## Abstract

Reservoir computing (RC) offers advantages in processing time‐series data with reduced training costs and simpler architectures. This study presents a hardware‐implemented RC system utilizing multifunctional memristors fabricated using a single process. By leveraging a ferroelectric‐based memristor (FM) as a volatile reservoir layer and a redox‐based memristor (RM) as a non‐volatile readout layer, seamless integration without additional fabrication steps is achieved. The dual‐functional memristor structure enables electrical conversion from FM to RM, enhancing system scalability and versatility. Comprehensive electrical measurements, including low‐frequency noise analysis and weight update linearity evaluation, validate the memristors’ performance. Potentiation and depression processes achieve a linearity factor improvement to ensure precise synaptic weight tuning, with cycle‐to‐cycle variation <2.3%. Additionally, the ferroelectric‐based memristor exhibits a cycle‐to‐cycle variation of 3.52%, maintaining distinct reservoir states with minimal overlap. Offline training demonstrates a high classification accuracy of 93.3% on the Modified National Institute of Standards and Technology dataset, while online training achieves an accuracy of 88.2% with incremental pulse schemes, surpassing the accuracy of identical pulse schemes (65.1%). These results establish the practical viability of multifunctional memristors for neuromorphic systems, establishing a robust foundation for next‐generation computing technologies

## Graphical abstract

Multifunctional memristors combining ferroelectric and redox switching are co‐fabricated via a single process, enabling compact, reconfigurable reservoir computing hardware. Ferroelectric mode offers tunable volatility for dynamic reservoirs, while redox mode provides stable multilevel weight storage. Electrical transformation enables full RC system implementation, achieving 93.3% offline and 88.2% online MNIST accuracy. This work offers a scalable, task‐adaptive architecture for neuromorphic AI applications.

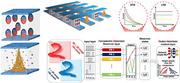

## 1 Introduction

Neural networks, inspired by how human brain neurons process and transmit information, trace their origins back to the perceptron model developed in the 1950s.^[^ ^1^ ^]^ Subsequent advancements, including the introduction of the multilayer perceptron (MLP) and backpropagation algorithms, enabled neural networks to solve nonlinear problems, addressing increasingly complex tasks.^[^ ^2^, ^3^ ^]^ However, traditional neural networks faced considerable challenges, particularly when processing long sequence data, owing to issues such as vanishing or exploding gradients, which hindered stable learning.^[^ ^4^, ^5^ ^]^ To overcome these limitations, reservoir computing (RC) emerged in the early 2000s as a novel neural network architecture framework. Unlike conventional networks that require training of all the weights, RC simplifies the learning process by keeping the weights of the dynamic reservoir layer fixed and training only the readout layer.^[^ ^5^, ^6^ ^]^ This approach reduces computational costs, mitigates gradient‐related issues, and excels in the processing of time‐series data owing to its ability to capture and store complex temporal patterns in the reservoir layer.^[^ ^5^, ^7^, ^8^ ^]^ Recent advances have focused on hardware‐based RC systems utilizing memristors. Memristors, with their tunable resistance states, compact size, low power consumption, and increased switching speeds, are ideal candidates for mimicking synaptic functions in neural networks.^[^ ^9^, ^10^, ^11^, ^12^, ^13^, ^14^ ^]^ By employing memristors in both the reservoir and readout layers, RC systems can enhance considerably their learning and processing capabilities. Memristors’ inherent ability to learn and store dynamic patterns allows for efficient reservoir layer construction, while their adjustable weights in the readout layer enable precise output tuning.^[^ ^15^, ^16^ ^]^ Building on prior research, we explore herein multifunctional memristors capable of exhibiting both ferroelectric polarization and filamentary resistance switching within a single device.^[^ ^13^ ^]^ This dual functionality, facilitated by the HfAlO (HAO) thin film, allows the device to transition from a ferroelectric memristor to a resistive random‐access memory (RRAM) through simple electrical manipulation. Leveraging this adaptability, we propose an RC system with a reservoir layer composed of a ferroelectric memristor and a readout layer of RRAMs, both fabricated from the same material and process. The ferroelectric memristor provides promising nonvolatile memory characteristics that are also influenced by depolarization‐induced ferroelectric degradation.^[^ ^17^, ^18^, ^19^ ^]^ Interestingly, this property enables dynamic RC behavior with virtual nodes in the reservoir layer. Meanwhile, RRAMs in the readout layer offer stable nonvolatile memory, allowing for tailored designs to meet application‐specific requirements. Our RC system achieves energy‐efficient, robust hardware implementation by connecting ferroelectric‐based reservoirs in parallel to an RRAM‐based binary readout layer. This configuration supports large‐scale parallel computation and facilitates the hardware realization of weight summations essential for artificial neural networks. The integration of memristors, with their physical and computational advantages, marks a major step forward in the development of future computing technologies.

**Figure 1:**
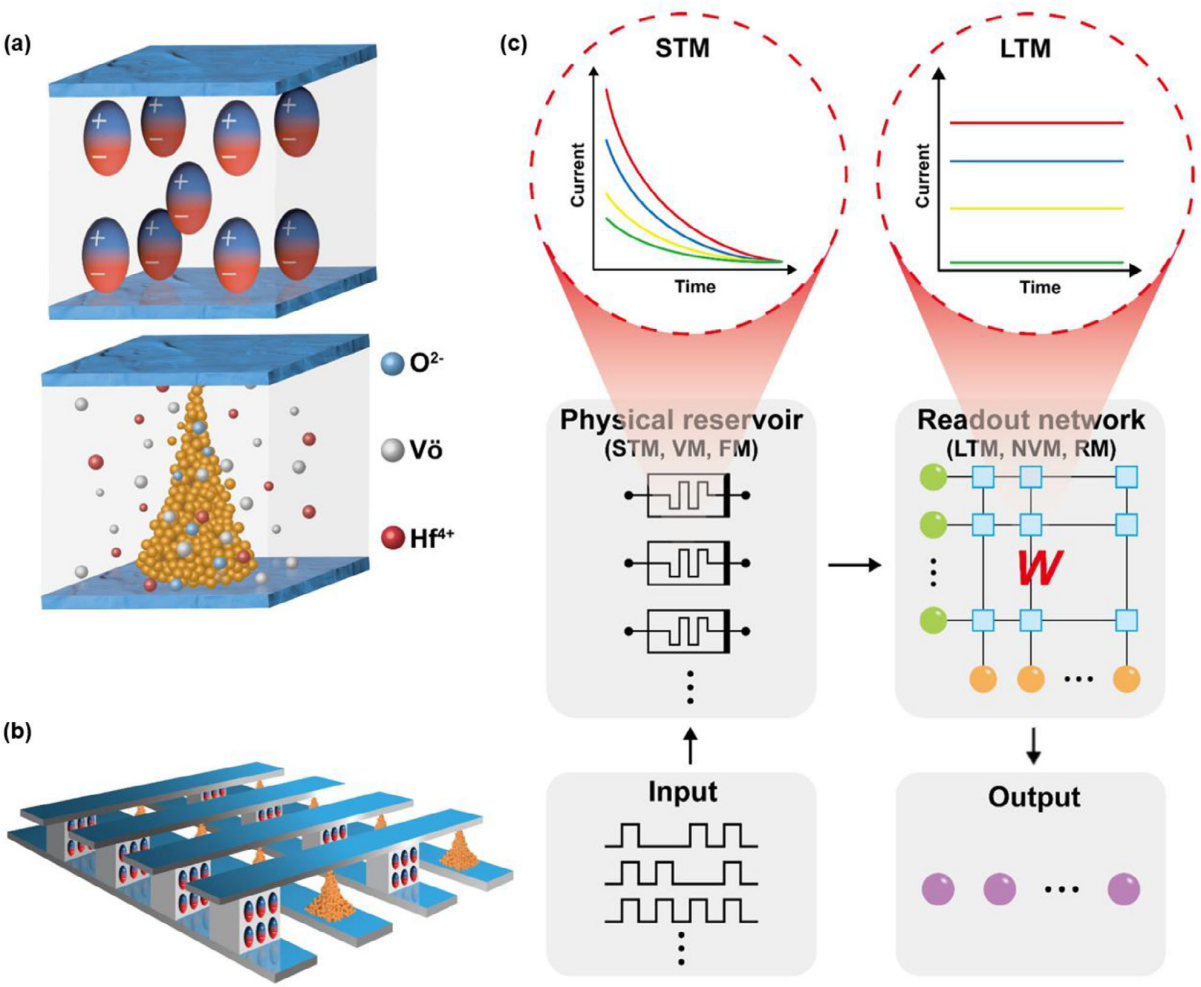
a) Resistive switching mechanisms: FM (top) switches via polarization direction, while RM (bottom) switches through redox reactions. b) An example configuration of memristors with distinct functionalities fabricated using a shared process. The functionality of each device can be determined postfabrication based on electrical stimulation, allowing flexibility based on the purpose of individual cells, layers in the stack, or specific areas within the wafer. c) Schematic representation of a hardware‐implemented reservoir computing (RC) system. The physical reservoir is composed of volatile memristors(VM), while the readout network utilizes nonvolatile memristors(NVM). Input data are encoded as a pulse train, processed through the physical reservoir (ferroelectric‐based memristors (FM)), and the synaptic weights are stored in the readout layer (redox‐based memristors (RM)).

In this study, we propose the co‐fabrication of ferroelectric‐ and redox‐based memristors for a hardware‐implemented RC system designed for tasks such as pattern recognition, time‐series prediction, and signal processing.^[^ ^20^, ^21^, ^22^ ^]^ The reservoir layer, constructed with ferroelectric‐based memristors (FMs), and a readout layer, composed of redox‐based memristors (RMs), share the same fabrication process and materials without requiring additional steps. The two memristors have distinctly different resistive switching mechanisms. Specifically, the FM is based on resistive switching according to the polarization direction (**Figure** **1**a, top), while the RM relies on resistive switching caused by redox reactions (Figure 1a, bottom). Postfabrication, the functionality of each cell is determined through electrical manipulation (Figure 1b). The depolarization characteristics of the FM enable the construction of a volatile reservoir layer, while the RM, converted through electrical stimulation, forms a nonvolatile readout layer requiring long‐term memory properties (Figure 1c). Note that while the transition from the ferroelectric memristor (FM) mode to the redox‐based memristor (RM) mode in our device is not fully reversible and proceeds as a one‐way conversion under specific conditions, this approach remains a practical and rational strategy for region‐specific device functionality within a device–system co‐design framework. We adopted the HAO/HfO_2_ structure as the optimal design based on preliminary investigations of device characteristics based on the interfacial layer (IL). Comprehensive electrical measurements, including low‐frequency noise analysis, ensured reliable characterization of the memristors. In the RRAM‐based readout layer, critical attributes, such as the linearity of weight updates and cycle‐to‐cycle (C2C) variation, were incorporated into pattern recognition simulations. While the ferroelectric‐based reservoir layer exhibited a C2C variation of less than 3.52%, the reservoir states remained distinct with minimal overlap, ensuring reliable operation. Our simulations showed only a 5.1% decrease in pattern recognition accuracy compared with offline learning, demonstrating the practicality of the proposed system. By enabling multifunctionality within a single memristor cell, our approach offers a versatile and efficient foundation for neuromorphic systems. This innovation sets a new benchmark for memristor‐based hardware, extending the applications of neuromorphic computing to meet the demands of diverse artificial intelligence research.

## 2 Results and Discussion

### 2.1 Device Fabrication and Material Characterization

**Figure 2:**
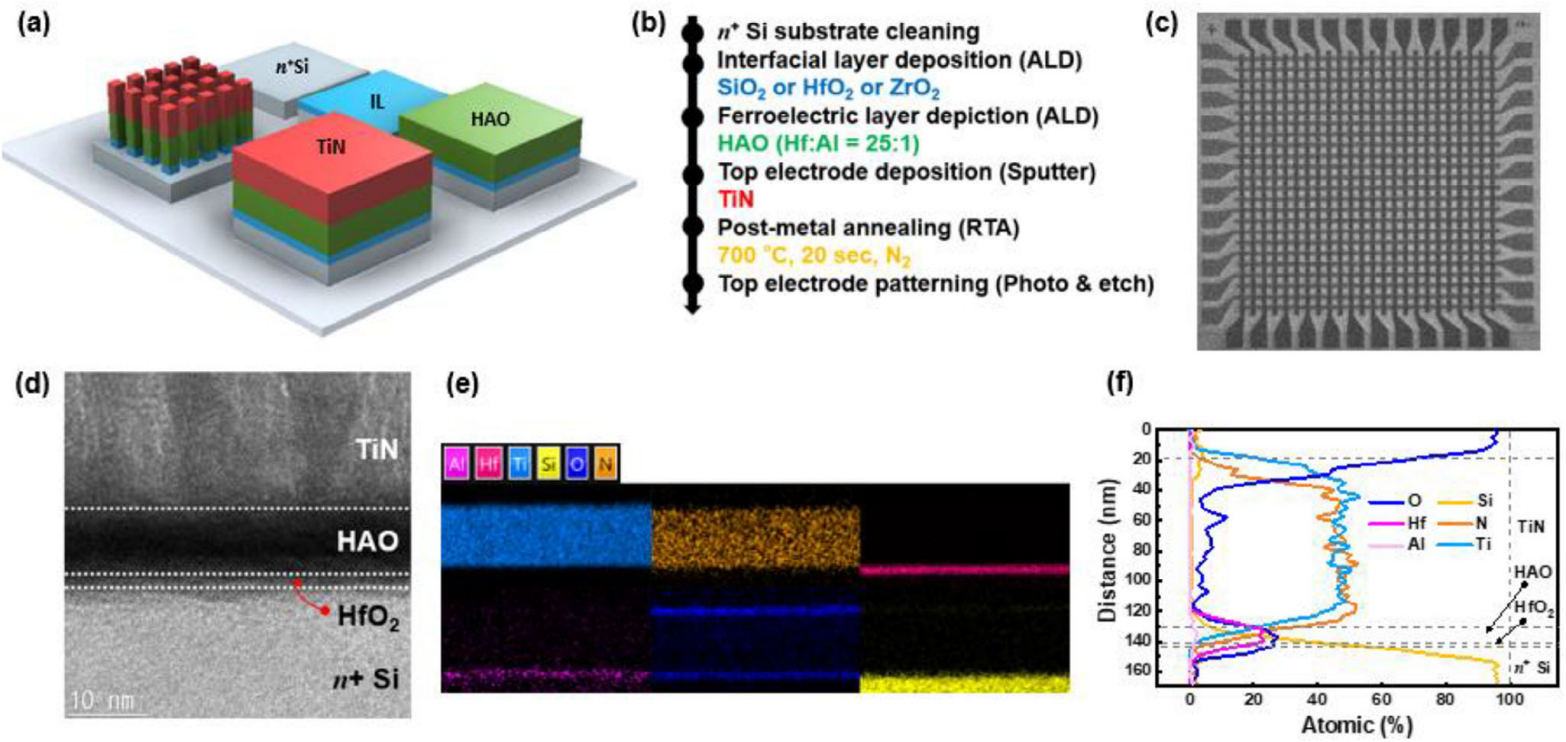
a) Illustration of the fabrication process for FM and RM. Both devices share the same manufacturing steps, highlighting compatibility and scalability. b) Simplified process flow. c) Scanning electron microscopy images of the fabricated 24×24 crossbar array. d) High‐resolution transmission electron microscopy image of the fabricated memristor, showing distinct layers, including the TiN, HAO, and n^+^ Si substrate. e) Energy‐dispersive X‐ray spectroscopy (EDS) color mappings of key elements (Al, Hf, Ti, Si, O, and N), providing elemental distribution within the device structure. f) EDS depth profile (aligned with the stacking direction of d and e), revealing the atomic composition across the device layers, ensuring precise material integration and uniformity in the fabrication process.

The fabrication process for the two memristors, which share identical processing steps, material compositions, and stack structures (TiN/HAO/HfO_2_/n⁺ Si), is described as follows. First, the heavily doped n⁺ Si substrate, which served as the bottom electrode (BE), underwent cleaning with a piranha solution to remove organic residues and other contaminants. Native oxide on the surface was then eliminated using a diluted HF solution to ensure a pristine silicon surface. Following the substrate preparation, a 1 nm HfO_2_ layer and a 9 nm HAO layer were sequentially deposited using atomic layer deposition (ALD). This process ensured excellent uniformity and precise control over the thickness of these functional layers. Subsequently, a 100 nm TiN layer, serving as the top electrode (TE), was deposited via sputtering. To induce ferroelectricity in the HAO film, postmetal annealing (PMA) was performed at 700 °C for 20 s in a nitrogen environment. Finally, the device structure was completed using photolithography and dry etching to pattern the TE. **Figure** **2**a presents a 3D schematic of the overall fabrication process, while Figure 2b outlines the main process steps. These illustrations highlight the simplicity and scalability of the fabrication procedure, which requires no additional processing steps for different memristor functionalities. Figure 2c presents the scanning electron microscopy image of the fabricated 24×24 crossbar array. Note that all electrical measurements discussed in this study were performed on individual cells with a shared bottom electrode configuration. Nevertheless, the proposed device structure is readily extendable to crossbar‐type arrays, as demonstrated in Figure 2c. The corresponding fabrication process for this array implementation is provided in Figure S1. The structural integrity of the fabricated device was evaluated using cross‐sectional transmission electron microscopy (TEM). Figure 2d shows TEM image of the TiN/HAO/HfO_2_/n⁺ Si stack, confirming that each layer is deposited with the intended thickness and uniformity. The thicknesses of the HAO and HfO_2_ layers were measured to be ≈9 and 1 nm, respectively, validating the precision of the ALD process. Elemental composition and distribution across the device structure were analyzed using energy‐dispersive X‐ray spectroscopy (EDS). Figure 2e displays the EDS color mappings for Ti, N, Hf, Al, O, and Si, providing a visual representation of the spatial distribution of these elements. Furthermore, Figure 2f shows the EDS depth profile, which illustrates the atomic percentages of these elements as a function of distance from the TiN TE to the n⁺ Si BE. This depth profile offers insights into the interfacial composition and potential material diffusion during the fabrication process. Interestingly, oxygen was detected in the TE region despite TiN's well‐known role as a capping layer to prevent oxidation.^[^ ^23^, ^24^ ^]^ This could be attributed to residual oxygen or moisture in the atmosphere during the sputtering process or subsequent experimental handling.^[^ ^25^ ^]^ Additionally, exposure to the atmosphere after the rapid thermal annealing (RTA) process may contribute to this phenomenon. High‐temperature processes like PMA can also promote interfacial reactions and diffusion of certain elements, leading to subtle compositional changes within the stack.

### 2.2 Ferroelectric‐Based Volatile Memristor: Physical Reservoir Layer

**Figure 3:**
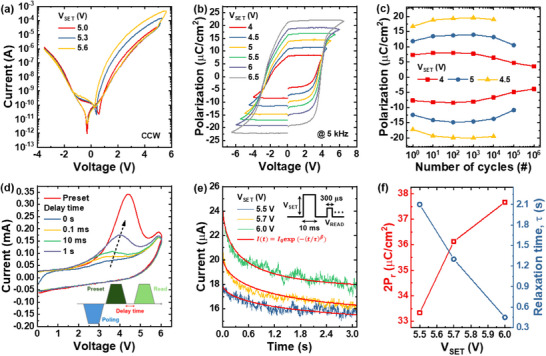
a) Hysteresis I–V curves for tunneling current under varying V_SET_ conditions, illustrating reliable tunneling electroresistance properties. b) Polarization–voltage (P–V) curves measured at 5 kHz, achieving a maximum 2P_r_ of 44.4 µC cm^−2^ at 6.5 V. c) Polarization cycling test, showing wake‐up, stable, and fatigue stages with breakdown after 10^5^ cycles at 5 V. d) Switching current measurements at various delay times, demonstrating polarization retention loss owing to depolarization fields. e) Transient current responses for different V_SET_ values, showing the volatile nature of the device with current‐decay fitted stretched exponential functions. f) Inverse relationship between 2P_r_ and relaxation time (τ), highlighting tunability of device performance for neuromorphic applications.

**Figure** **3**a presents the hysteresis I–V curves obtained from direct current (DC) double‐sweep measurements of the fabricated FM device at different set voltages (V_SET_). The tunneling barrier changes depending on the direction and magnitude of the applied electric field, and the unique properties of the ferroelectric devices are quantified by the tunneling electroresistance (TER) ratio. The TER ratio increases at higher V_SET_ values owing to voltage‐controlled partial polarization switching, a characteristic of ferroelectric devices.^[^ ^26^, ^27^, ^28^ ^]^ This tunable TER property offers several advantages for neuromorphic computing applications.^[^ ^29^, ^30^ ^]^ For instance, as a nonvolatile device, ferroelectric devices can exhibit multiple conductance states based on the input signal. Conversely, as a volatile device, such as in our study, the degree of polarization relaxation can be dynamically controlled by adjusting the input signal parameters. Preliminary investigations comparing devices with HfO_2_, SiO_2_, and ZrO_2_ ILs revealed that the TiN/HAO/HfO_2_/n⁺ Si structure exhibits superior uniformity and a higher TER ratio across 20 randomly selected cells (Figure S2 in the Supporting Information). Furthermore, the TER ratio is plotted in Figure S3 for varying V_read_ conditions, achieving a TER ratio of ≈72.4 at a V_read_ value of 1.2 V. Moreover, the device exhibits intrinsic self‐rectifying behavior, which effectively suppresses sneak path currents in crossbar arrays and enables a 10% read margin at Vset = 5.6 V for arrays up to 194 × 194 in size (Figure S4 in the Supporting Information). Figure 3b illustrates the polarization–voltage (P–V) curves measured using the positive‐up‐negative‐down method at 5 kHz. As V_SET_ increases, the remanent polarization (P_r_) also increases, confirming partial polarization switching. At a V_SET_ of 6.5 V, the maximum 2P_r_ value reaches 44.4 µC cm^−2^, comparable to other ferroelectric materials based on HfO_2_.^[^ ^31^, ^32^, ^33^ ^]^ This result demonstrates the robustness of the HAO layer in our devices and its applicability to ferroelectric memory applications. The reliability of the FM device was further assessed based on polarization cycling tests, as shown in Figure 3c. Similar to other ferroelectric two‐terminal devices, such as ferroelectric tunnel junctions or ferroelectric diodes, the device exhibits distinct wake‐up, stable, and fatigue stages. These results highlight the stability of the FM device under repeated operation conditions and the trends observed in ferroelectric cycling reliability. To examine the retention characteristics of the FM device, polarization switching currents were measured by varying the delay times. The inset of Figure 3d illustrates the measurement sequence: a prepoling pulse aligns the polarization in one direction, followed by a preset and a read pulse to measure the switching current. Detailed pulse information is provided in Figure S5. Figure 3d shows that when the delay time is zero, no additional switching current is recorded, as all domains are aligned. However, as the delay time increases, the switching current reappears and gradually returns to its initial state, indicating the randomization of aligned domains over time due to depolarization fields.^[^ ^34^, ^35^, ^36^, ^37^ ^]^ This phenomenon is a known limitation of ferroelectric devices with metal–ferroelectric–insulator–semiconductor (MFIS) structures but can be advantageous for neuromorphic applications where volatile characteristics are required. Figure S5 further illustrates the 2P_r_ values as a function of delay time, confirming polarization retention loss. To validate the volatile nature of our FM device and identify controllable parameters, transient current measurements were performed under varying V_SET_ conditions, as shown in Figure 3e. The device current increases at higher V_SET_ values but decays rapidly to its initial state once the set pulse is removed. The decay rate accelerates with increasing V_SET_, confirming the volatile behavior of the device. The current decay curves fit well with the stretched exponential function,^[^ ^38^ ^]^ which describes electronic or chemical relaxation phenomena, as indicated by the red fitting lines. These nonequilibrium physical phenomena are extensively observed in semiconductor devices.^[^ ^39^, ^40^ ^]^ The fitting parameters are listed in Table S1 in the Supporting Information. Figure 3f depicts the relationship between the 2P_r_ value and the relaxation time constant (τ) as a function of V_SET_. An inverse correlation is observed: as V_SET_ increases, 2P_r_ increases while τ decreases. This relationship can be attributed to the depolarization field, described by the following equation:^[^ ^35^, ^36^ ^]^

(1) $$E_{dep}=\frac{-Pd_{int}}{\varepsilon_{0}\left(d_{int}\varepsilon_{FE}+d_{FE}\varepsilon_{int}\right)}$$

where *E_dep_* is the depolarization field, *P* is the ferroelectric polarization surface charge, *d_int_* and *d_FE_* are the thicknesses of the interface and ferroelectric layers, respectively, and ε_0_, ε_*FE*_, and ε_*int*_ are the permittivities of the vacuum, ferroelectric layer, and the interface layer, respectively. As the V_SET_ increases, *P* increases, leading to a stronger depolarization field and smaller τ values, which accelerate the volatility of the device. Our FM device, with relaxation time constants of the order of seconds, demonstrates promising characteristics for dynamic RC. By tuning the input signal conditions (e.g., amplitude, width) and process parameters (e.g., IL thickness), the reservoir layer can be optimized for specific applications, paving the way for enhanced data‐processing speeds in RC systems. Additionally, the observed volatility of our FM device is likely attributed to a combination of physical mechanisms, including material defects, charge‐trapping effects, and transient capacitive charging. A comprehensive discussion of these origins is provided in Note S2.^[^ ^41^, ^42^, ^43^, ^44^, ^45^, ^46^, ^47^ ^]^

### 2.3 Redox‐Based Nonvolatile Memristor: Readout Layer

**Figure 4:**
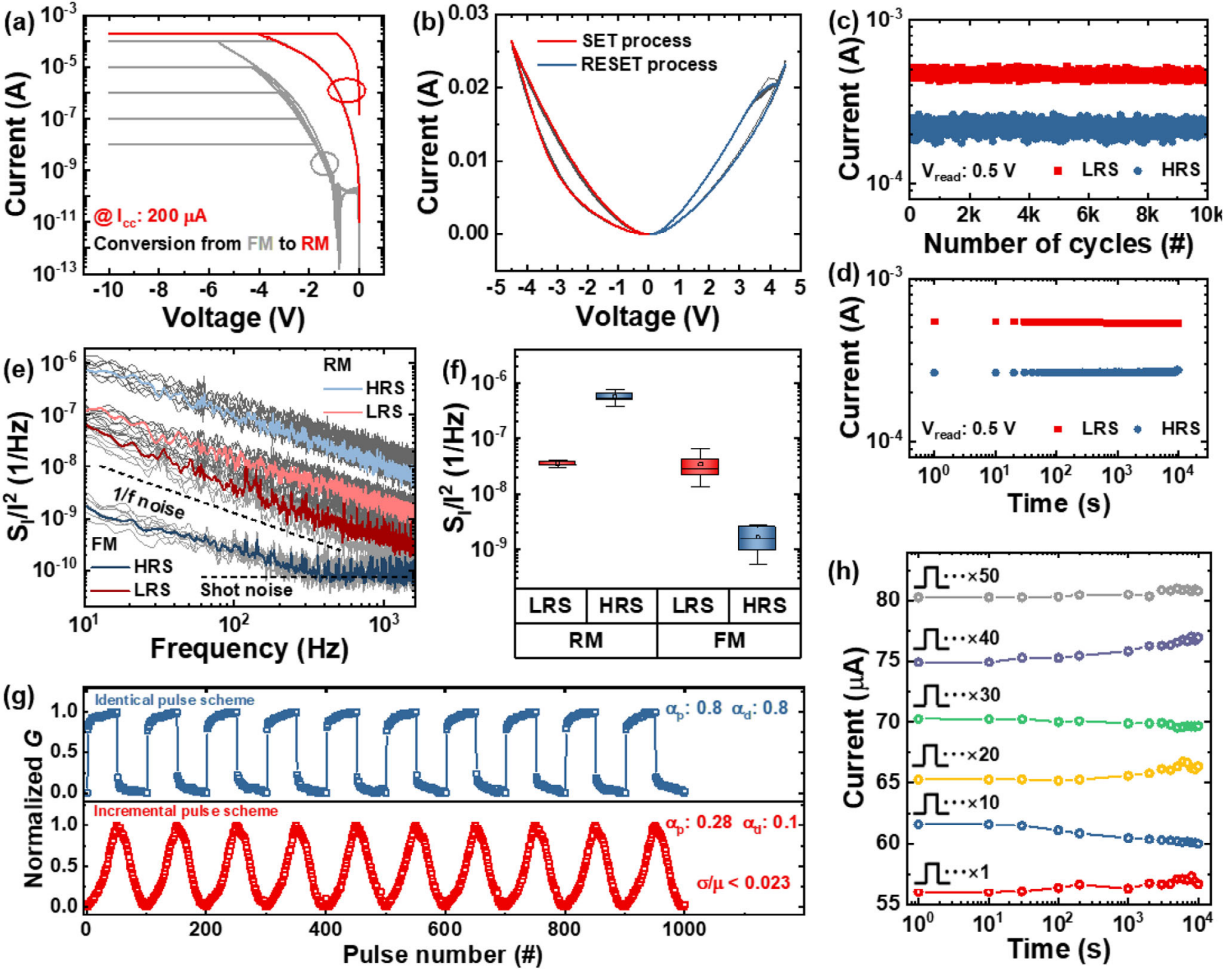
a) Transition from FM to RM under a compliance current of 200 µA. b) Direct current I–V double sweeps showing interface‐type resistive random‐access memory characteristics after the FM‐to‐RM transition. c) Stable switching performance with over 10000 cycles and d) data retention exceeding 10^4^ s. e) Comparison of normalized current power spectral density (S_I_/I^2^) for FM and RM devices, highlighting distinct noise characteristics. f) Statistical validation of noise trends. g) Long‐term potentiation/long‐term depression characteristics of the RM device using identical (Top) and incremental (Bottom) pulse schemes, with improved linearity and consistent operation. h) Retention characteristics of the incremental pulse scheme, confirming stability up to 10^4^ s.

To transition from FM to RM within the same cell, a well‐established forming process is required. In our device, which includes two dielectric layers, the initial hard breakdown occurs in the HfO_2_ layer owing to its smaller thickness. This is followed by the formation of incomplete conductive paths within the HAO film. When subjected to a sufficiently high‐electric field, oxygen vacancies, and ions are generated and migrate between the top interface and the bulk of the dielectric layers. The polarity of the applied voltage governs the formation and dissociation of oxygen vacancies, resulting in redox reactions that drive the resistive switching behavior of the RM device. The detailed mechanism of RM operation and current conduction is elaborated in Figure S6. **Figure** **4**a demonstrates the transition from FM to RM. At a compliance current (I_cc_) of <100 µA, FM behavior persists, while an I_cc_ of 200 µA triggers the transition to RM. Once transitioned to RM, the device exhibits typical interface‐type RRAM characteristics, as shown in the DC I–V double sweeps in Figure 4b. Although the on/off ratio is modest, the device exhibits exceptionally stable switching performance, achieving over 10000 repetitive switching cycles and data retention exceeding 10^4^ s, as shown in Figure 4c,d, respectively. Process optimizations, such as precise thickness control, doping, or the incorporation of interlayers, could further improve the on/off ratio.^[^ ^48^, ^49^, ^50^ ^]^ To differentiate the two devices, we analyzed their noise characteristics, which are unique to each semiconductor device. Noise analysis provides insights into defect states, the primary cause of noise, and helps elucidate conduction mechanisms.^[^ ^51^, ^52^, ^53^, ^54^ ^]^ Figure S7 provides detailed descriptions of the noise measurement methodology. Figure 4e compares the normalized current power spectral density (S_I_/I^2^) in the low‐resistance state (LRS) and high‐resistance state (HRS) for both FM and RM devices, using data from 10 randomly selected cells per resistance state. For FM, the noise power in the LRS is approximately 100 times greater than that in the HRS, while RM exhibits the opposite trend: the noise power in the LRS is approximately 10 times smaller than that in the HRS. These observations are statistically validated in Figure 4f, which highlights the distinct operational and conduction mechanisms of FM and RM. This analysis confirms that the transition process fundamentally alters the intrinsic device characteristics: FM operates via polarization switching,^[^ ^13^, ^32^, ^55^ ^]^ while RM is driven by redox reactions.^[^ ^54^, ^56^, ^57^ ^]^ Figure 4g explores the long‐term potentiation (LTP) and long‐term depression (LTD) characteristics of the RM device over 10 cycles. The linearity of weight updates and C2C variation are critical parameters for pattern recognition simulations in RC systems.^[^ ^58^, ^59^, ^60^ ^]^ To improve these characteristics, two pulse schemes were evaluated. The information on the applied pulse schemes is provided in Figure S8. In the top panel of Figure 4g, identical pulses are applied to achieve 50 conductance states. However, the RM device exhibits abrupt resistance changes when identical pulses are used, leading to an initial offset that increases the nonlinearity of weight updates and limits the achievable conductance states. To address this, an incremental pulse scheme was implemented. The bottom panel of Figure 4g demonstrates the LTP/LTD characteristics under this scheme, showing significant improvements in linearity. The nonlinearity factor for potentiation (α_*p*_) improved from 0.8 to 0.28, while the factor for depression (α_*d*_) improved from 0.8 to 0.1. Additionally, C2C variation (σ/µ) over 10 cycles remained below 0.023, ensuring consistent and reliable operation. Figure 4h illustrates the retention characteristics of the RM device as a function of the number of input pulses (incremental pulse scheme). Despite minor current variations, the device demonstrates stable retention up to 10^4^ s, confirming its suitability for use as a nonvolatile memory in RC systems. These results demonstrate that, although the redox‐based memristor exhibits intrinsic nonlinearity in conductance modulation under identical pulse conditions, this limitation can be effectively mitigated through a one‐time incremental pulse programming strategy. This approach is fully compatible with the offline training paradigm of reservoir computing and ensures stable and precise weight initialization without imposing additional real‐time computational overhead.

### 2.4 Precise Weight Adjustment in RC Systems Using the Incremental Step Pulse with Verify Algorithm (ISPVA)

**Figure 5:**
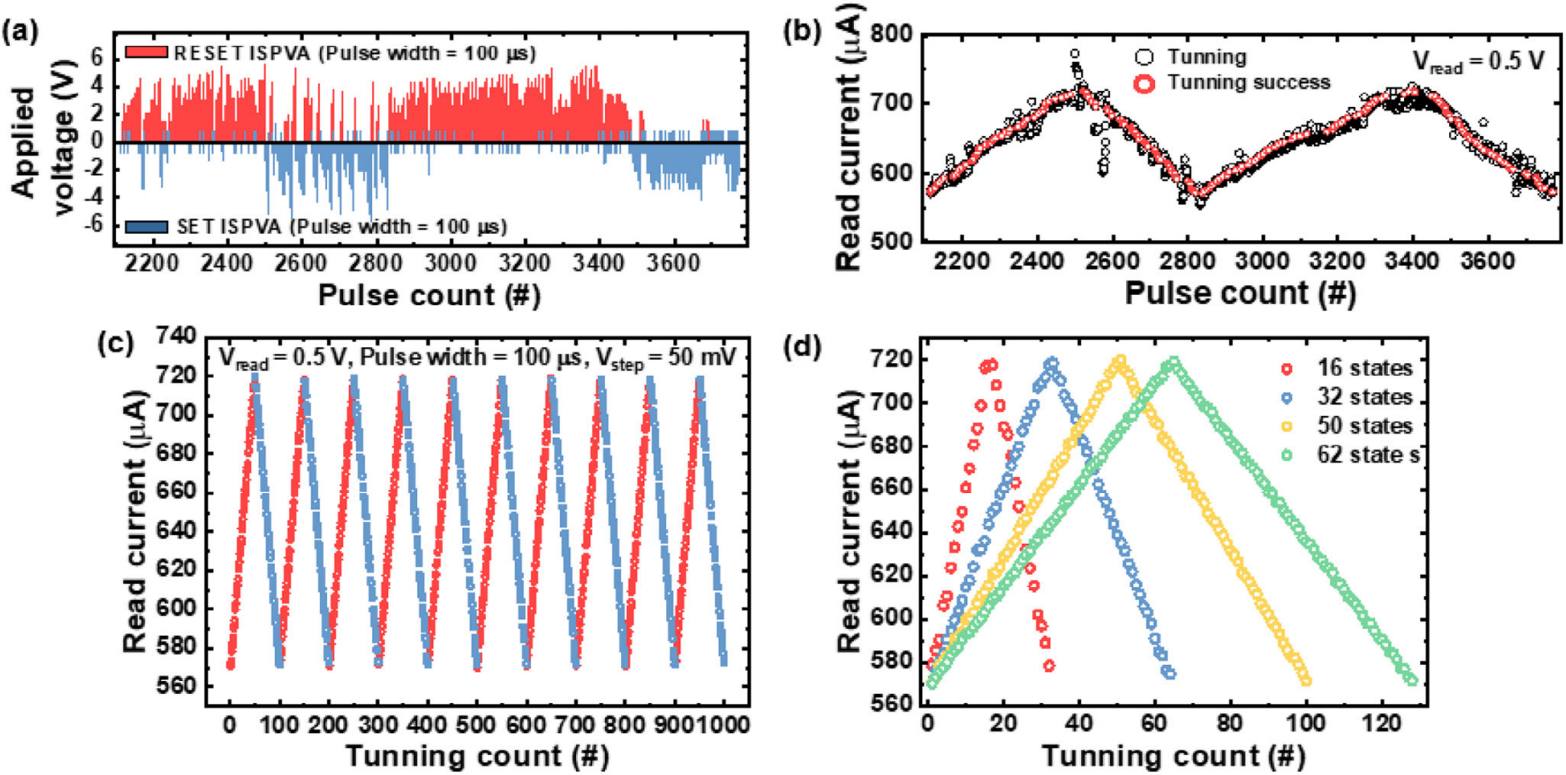
a) Detailed incremental step pulse with verify algorithm (ISPVA) process for target weight tuning, utilizing set and reset pulses with a width of 100 µs and an incremental pulse amplitude step size of 50 mV. b) Representative example of successful weight tuning, demonstrating precise adjustment using iterative feedback. c) Stable weight tuning over 1000 consecutive tuning cycles for 50 states, highlighting robustness and repeatability. d) Implementation of multibit target weights, demonstrating the RM device's capability for high‐resolution weight tuning for neuromorphic computing.

In neuromorphic systems, artificial neural networks rely on the adjustment of the weight values of artificial synapses within the network to perform desired functions.^[^ ^9^, ^16^, ^61^ ^]^ This weight adjustment process is achieved through learning, which can be classified into two approaches: online and offline training. In offline training, pretrained optimal weights are programmed onto artificial synapses (physical devices) before their use in the system. Conversely, online training involves real‐time learning directly on the physical device, where weights are dynamically updated during operation.^[^ ^60^, ^62^, ^63^ ^]^ Both approaches have distinct advantages and limitations, with the choice depending on the system environment and application requirements. To explore the weight‐tuning performance of the RM device in offline training, the ISPVA was employed. This method enables precise weight adjustments and evaluation of multibit characteristics.^[^ ^64^, ^65^ ^]^ **Figure** **5**a illustrates the detailed ISPVA process for target weight tuning. The set and reset pulses used in the ISPVA process have an identical pulse width of 100 µs, and the pulse amplitude increment step size was set to 50 mV. During the ISPVA process, a read pulse is applied between each input pulse to inspect the current device state and verify whether it falls within the target current range, defined as 100 nA with a tolerance of ±50 nA. This iterative feedback mechanism ensures precise tuning of the device to the desired weight. The results confirm that the RM device can successfully achieve target weight tuning for 50 states, as demonstrated in Figure 5b. Furthermore, as shown in Figure 5c, stable weight tuning was achieved over 1000 consecutive tuning cycles, highlighting the device's robustness and repeatability. Increasing the number of bits per cell is a promising strategy to enhance the classification accuracy of neural networks. Figure 5d demonstrates the successful implementation of target weights corresponding to 4 bits (16 states), 5 bits (32 states), and up to 6 bits (64 states), even with the device's limited on/off ratio. These results showcase the capability of the RM device to achieve high‐resolution weight tuning, which is crucial for improving system performance in neuromorphic computing. In summary, the experimental results indicate that the RM device is highly suitable for offline (ex‐situ) learning applications. The device can accurately and reliably transfer target weights to synapses, providing a solid foundation for its use as a synaptic element in neuromorphic systems.

### 2.5 Implementation of Reservoir Computing System using multifunctional memristors

**Figure 6:**
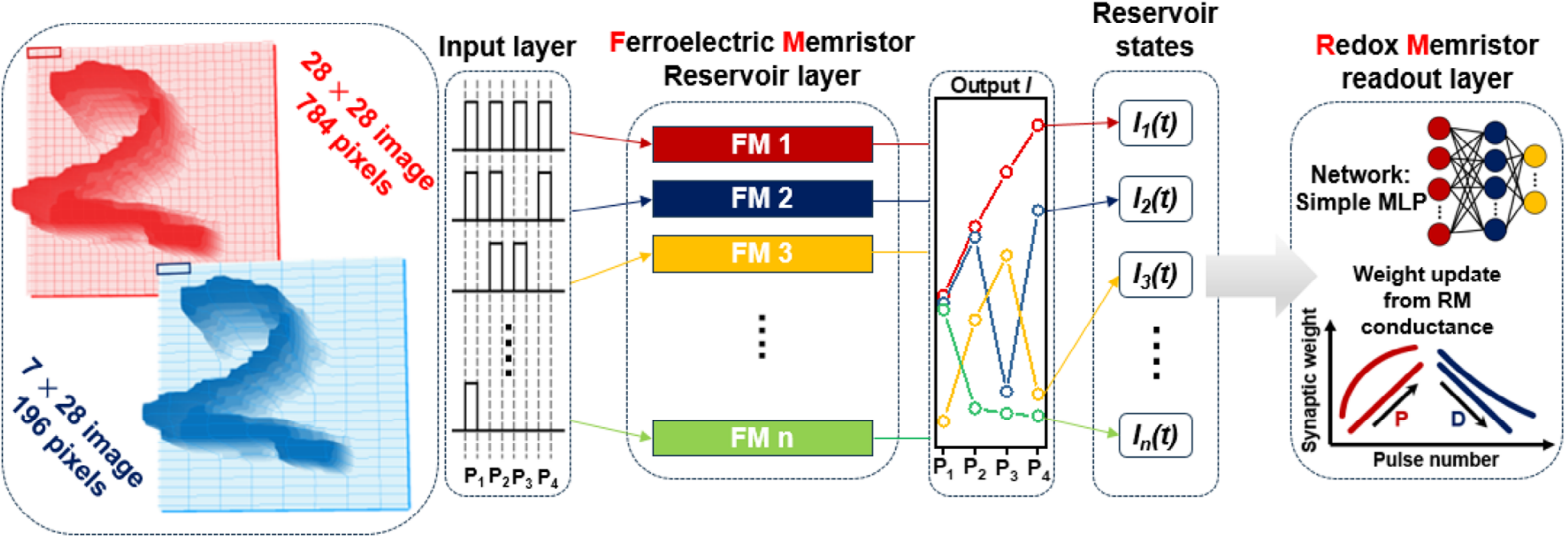
Conceptual framework of the RC system implemented using hybrid memristor devices for handwritten digit classification. The input layer processes binarized MNIST data into pulse trains (P_1_–P_4_), which are transmitted to the ferroelectric memristor‐based reservoir layer. The reservoir states evolve dynamically and are passed to the redox memristor‐based readout layer, where a simple multi‐layer perceptron (MLP) network predicts digits from 0 to 9.

Reservoir computing (RC) systems offer several advantages, including rapid learning, reduced network size, and lower training costs, making them well‐suited for processing various temporal and sequential information such as prediction, anomaly detection, and pattern recognition.^[^ ^6^, ^7^, ^8^, ^66^, ^67^ ^]^ For these reasons, many research groups have explored implementing RC systems using artificial synapses.^[^ ^15^, ^16^, ^20^, ^21^, ^22^ ^]^ In this study, we developed a hybrid device capable of selectively implementing both volatile and non‐volatile functionalities within a single cell, sharing the same fabrication process and materials. Utilizing this device, we demonstrated the efficient hardware implementation of an RC system. **Figure** **6** illustrates the preprocessing steps and conceptual framework for performing a handwritten digit classification task using the developed hybrid device. First, the input layer receives data from the Modified National Institute of Standards and Technology (MNIST) dataset. To simplify the data, the 784 grayscale pixel values (28 × 28) of each image are binarized: pixel values between 0 and 127 are assigned a value of 0, while values between 128 and 255 are assigned a value of 1. The binarized pixels are grouped into 196 groups (7 × 28), with each group represented as a pulse train (P_1_–P_4_). The states of the volatile physical reservoir layer evolve over time, and the states after the final pulse (P_4_) are transmitted to the readout network. The readout layer consists of 196 neurons that undergo a training process. These neurons are connected to a hidden layer comprising 100 neurons, which facilitates feature learning and mapping. The ultimate goal of the readout network is to predict digits from 0 to 9 based on the learned features. The MNIST dataset contains 60 000 images, of which 50 000 were used for training the model, including the reservoir layer, and the remaining 10 000 images were used to test the trained reservoir system.

**Figure 7:**
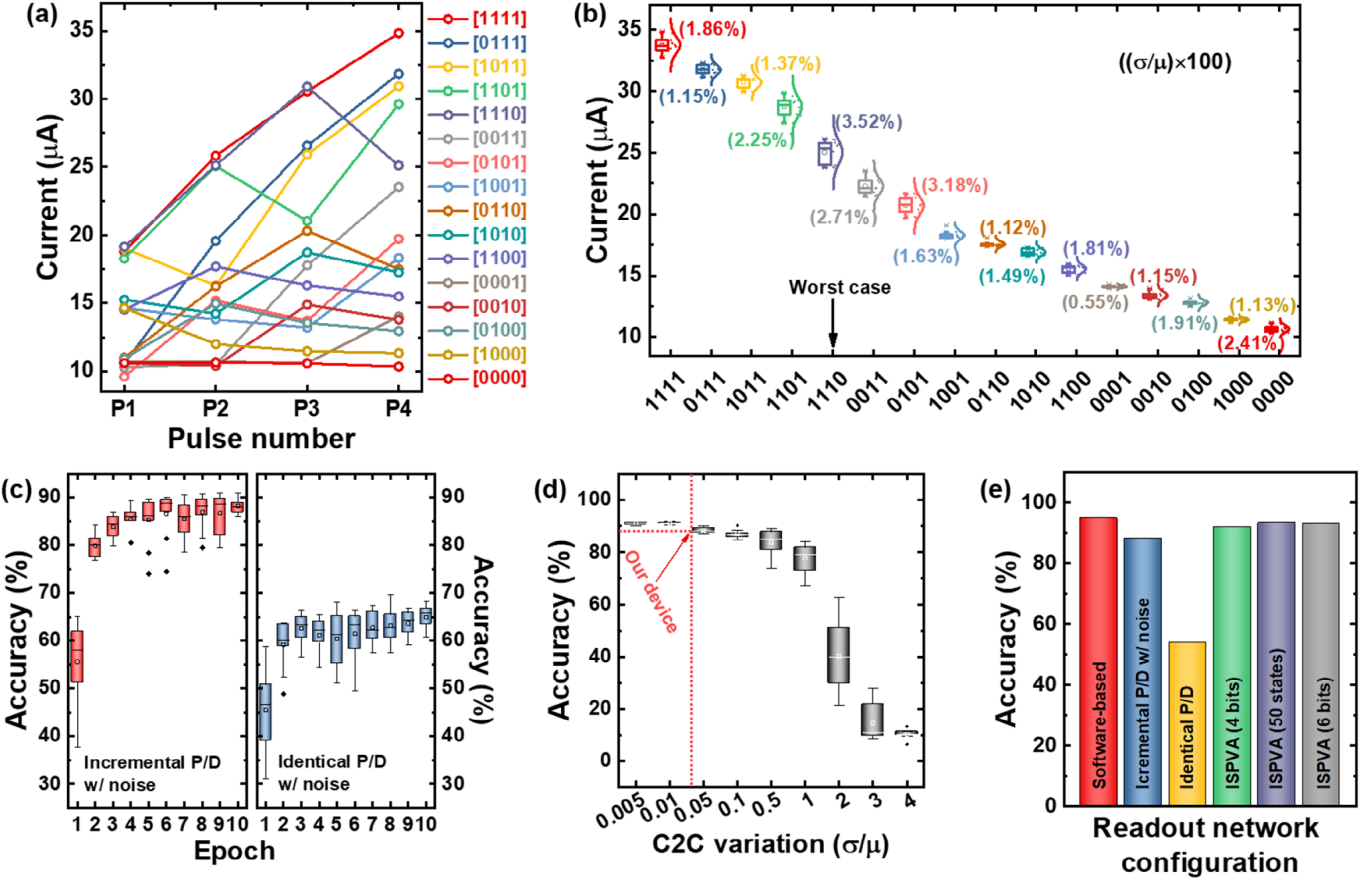
a) Current responses of FM devices to 16 different input pulse trains, successfully distinguishing 16 reservoir states. b) Cycle‐to‐cycle (C2C) variability analysis, demonstrating excellent stability with a maximum variability of 3.52%. c) Digit recognition accuracy based on FM and RM device data over 10 training epochs, highlighting the superior performance of the incremental pulse scheme (88.2%) compared with the identical pulse scheme (65.1%). d) Simulation of C2C variability effects on accuracy, showing our device's high tolerance. e) System performance in different readout network configurations.

**Figure** **7**a shows the current responses of FM devices to 16 different input pulse trains at each pulse stage (P_1_–P_4_). A pulse train with an amplitude of 5.4 V and a width of 10 ms was applied. After applying the final pulse, the 16 reservoir states were successfully distinguished, with excellent C2C variability of up to 3.52%, as shown in Figure 7b. Detailed current responses of FM devices measured over 10 repeated cycles are provided in Figure S9. Variations across 10 randomly selected FM devices were also evaluated, with results presented in Figure S10. Figure 7c presents the results of the digit recognition task based on measurement data from FM and RM devices over 10 training epochs. A major accuracy difference was observed depending on the pulse sequence applied for RM weight updates. As expected, the incremental pulse scheme achieved a higher accuracy of 88.2%, compared with 65.1% with the identical pulse scheme, owing to improved linearity in weight updates (see Figure 4g). C2C variability is typically recognized as a reliability issue in semiconductor devices, particularly in memristors, where conductance changes are highly random and difficult to predict. To investigate the robustness of the system implemented with our device, we simulated the effects of C2C variability, as shown in Figure 7d. The simulation assumes a Gaussian distribution for device variability with specified mean and standard deviation values, which were adjusted during the weight update process. The results show that accuracy decreases abruptly when the variability exceeds the value of one. However, the variability of our device was 0.023, ensuring high system accuracy and excellent variability tolerance (red line in Figure 7d). Figure 7e summarizes the system's performance in different readout network configurations. The accuracy of a software‐based system was 95%, while the accuracy of the system based on our device's experimental data was 88.2%. However, by optimizing the incremental pulse schemes, the accuracy was improved considerably (53.7%→88.2%). Additionally, implementing the system with offline training achieved an accuracy of 93.3% (with 50 conductance states, identical to online training). A representative confusion matrix is shown in Figure S11, demonstrating the accurate classification of handwritten digits from 0 to 9 in both online and offline training. These results validate the superior accuracy and scalability of our device.

## 3 Conclusion

In this study, we successfully developed multifunctional memristors capable of achieving both volatile and nonvolatile functionalities within a single cell using a shared fabrication process. By leveraging this innovative approach, we demonstrated the seamless integration of FM and RM devices into a hardware RC system, enabling efficient and scalable operation. The unique ability to convert FM and RM functionalities electrically without additional processing steps highlights the versatility of the proposed device architecture. Extensive material and structural analysis confirmed the integrity of the device stack (TiN/HAO/HfO_2_/n^+^ Si), while a variety of electrical measurements validated its performance. The I–V characteristics established stable switching behavior, while noise analysis provided insights into the conduction mechanisms and operational differences between FM and RM devices. The ISPVA facilitated precise multibit weight tuning, demonstrating robust and repeatable operation and achieved up to 6 bits (64 states) per cell, even with a limited on/off ratio. The system's applicability was further evaluated using both offline and online training approaches. Offline training achieved a classification accuracy of 93.3% for handwritten digit recognition tasks using the MNIST dataset, leveraging pretrained weights programmed onto physical synapses. Conversely, online training, achieved a maximum accuracy of 88.2% using incremental pulse schemes for RM weight updates, considerably outperforming identical pulse schemes (65.1%) owing to enhanced weight adjustment linearity. The robustness of the system was highlighted by its excellent C2C variability tolerance, with a measured variability of 0.023, ensuring consistent and reliable performance. This study highlights the immense potential of multifunctional memristors in advancing neuromorphic computing and establishes a solid foundation for their broader adoption in next‐generation computing technologies.

## 4 Experimental Section

### Device Fabrication

The fabrication process began with a highly doped n^+^ Si substrate (resistivity: 0.005 Ω·cm), which was cleaned using a 4:1 mixture of concentrated sulfuric acid (H_2_SO_4_) and hydrogen peroxide (H_2_O_2_) to remove heavy organic contaminants from the wafer surface. The native oxide layer and metallic impurities were subsequently removed using diluted HF. Following this, a 1 nm HfO_2_ interfacial layer and a 9 nm HAO ferroelectric layer were deposited onto the silicon substrate via ALD at a substrate temperature of 350 °C, using TEMAH and TMA as the precursors for HfO_2_ and Al_2_O_3_, respectively. Ozone was employed as the oxidant for both high‐k dielectric layers. The deposition rates for Al_2_O_3_ and HfO_2_ were 0.88 and 1.17 Å cycle^−1^, respectively, with one Al_2_O_3_ cycle following every 25 HfO_2_ cycles. This deposition ratio of 25:1 (3% Al content) was repeated three times to produce a total of 9 nm of HAO ferroelectric film. Subsequently, presputtering was conducted for 15 min to prevent contamination of the Ti target. A TiN layer (thickness = 100 nm) was deposited via sputtering (Endura 5500, AMAT) at a pressure of 3 mTorr, DC power of 5 kW, and a stage temperature of 200 °C and served as the top electrode. The flow rates of Ar and N_2_ gases were 15 sccm and 85 sccm, respectively. Postdeposition, the device underwent RTA, KVT‐3006T) at 700 °C for 20 s. Finally, TiN top electrodes with dimensions of 100 × 100 µm^2^ were patterned using photolithography and reactive ion etching.

### Electrical Measurements

The fabricated devices were characterized based on electrical measurements. DC I–V curves were measured using a semiconductor parameter analyzer (Keithley 4200‐SCS), while pulse measurements were performed with a 4225‐pulse measure unit (ultrafast module). The low‐frequency noise power spectral density was obtained using a low‐noise current amplifier (SR570) and a signal analyzer (35670A). The voltage applied to the top electrode was supplied by a semiconductor parameter analyzer (B1500A), and the device's output current was connected to the SR570 to convert current fluctuations into voltage fluctuations. The dynamic signal was then processed by the signal analyzer to calculate the power spectral density. The noise floor of the measurement system was approximately 10^−24^ A^2^ Hz^−1^, which was considerably lower than the noise level of the devices, ensuring that the measured power spectral density accurately represented the devices’ noise characteristics without interference from the system's noise.

## Conflict of Interest

The authors declare no conflict of interest.

## Supporting information



Supporting Information

## Data Availability

The data that support the findings of this study are available from the corresponding author upon reasonable request.
