# Supplementary material for: Integrated Design of Electrically Configurable Ferroelectric and Redox‐Based Memristors for Hardware‐Implemented Reservoir Computing
Source: Adv Sci (Weinh). 2025 Jun 10;12(33):e05688. doi: 10.1002/advs.202505688 (PMC12412490; doi:10.1002/advs.202505688)
Supplement: Supplementary file 1 — Supporting Information [file ADVS-12-e05688-s001.docx]

Supporting Information

Integrated Design of Electrically Configurable Ferroelectric and Redox-based Memristors for Hardware-Implemented Reservoir Computing

Jung-Kyu Lee, Yongjin Park, Euncho Seo, Jong-Ho Lee, Sungjoon Kim, and Sungjun Kim*

J.-K. Lee,

Department of Semiconductor Engineering, Gyeongsang National University, Jinju, Gyeongnam 52828, Republic of Korea

J.-H. Lee

Department of Electrical and Computer Engineering and Inter-university Semiconductor Research Center, Seoul National University, Seoul 08826, Republic of Korea

S. Kim

Department of AI Semiconductor Engineering, Korea University, Sejong 30019, Republic of Korea

Y. Park, E. Seo, S. Kim

Division of Electronics and Electrical Engineering, Dongguk University, Seoul 04620, South Korea
E-mail: sungjun@dongguk.edu

Keywords: reservoir computing; multifunction; ferroelectric; memristor; Hafnia


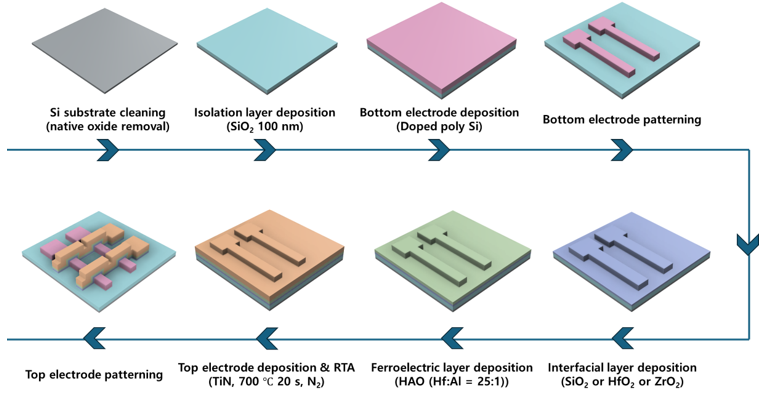


Figure S1. Figure S1. Fabrication process flow of the 24×24 ferroelectric memristor crossbar array, including n⁺ poly-Si bottom electrode, HAO ferroelectric layer, and TiN top electrode. The shared process enables electrical configurability of FM and RM modes within a single integrated array.


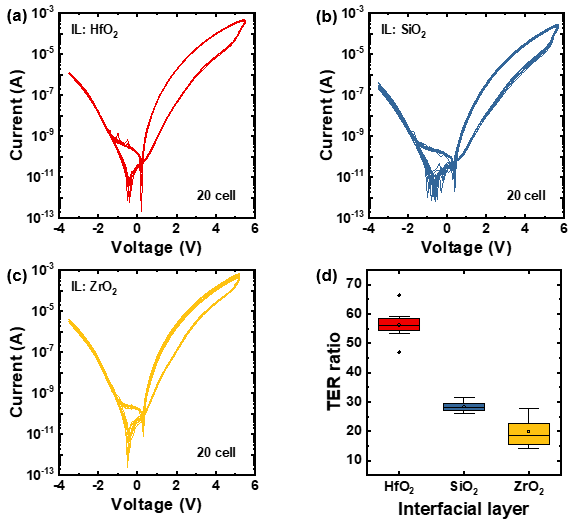


Figure S2. Comparative electrical characteristics of devices with different interfacial layers (ILs): a) TiN/HAO/HfO_2_/n⁺ Si structure, b) TiN/HAO/SiO_2_/n⁺ Si structure, and c) TiN/HAO/ZrO_2_/n⁺ Si structure, each showing representative DC I‒V curves measured across 20 randomly selected cells. The TiN/HAO/HfO_2_/n⁺ Si structure demonstrates superior uniformity in hysteresis behavior and enhanced TER ratio compared to devices with SiO_2_ and ZrO_2_ interfacial layers. (d) TER ratio distribution for the three interfacial layers, highlighting the significant performance advantage of HfO_2_ in achieving higher TER ratios and better uniformity across the tested cells. These results establish HfO_2_ as the optimal interfacial layer for the integration of ferroelectric-based volatile and non-volatile devices.


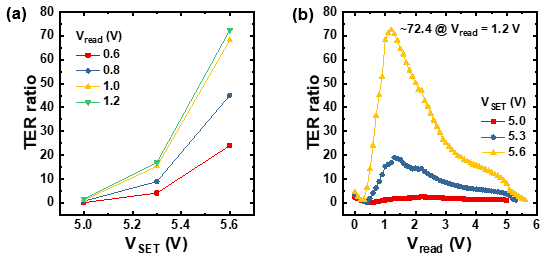


Figure S3. TER ratio as a function of a) V_SET_ at various V_read_ conditions (0.6 V, 0.8 V, 1.0 V, and 1.2 V) and b) V_read_ at different V_SET_ conditions (5.0 V, 5.3 V, and 5.6 V) for the fabricated device. The TER ratio increases significantly with higher V_SET_ and V_read_ values, achieving a maximum TER ratio of ~72.4 at a V_read_ of 1.2 V and V_SET_ of 5.6 V. These results demonstrate the strong dependence of the TER ratio on the applied voltage parameters, enabling fine-tuning of device performance.


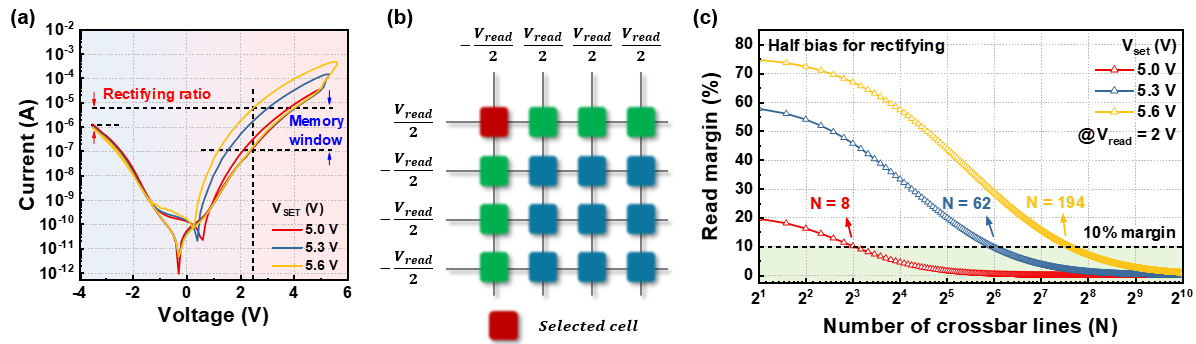


Figure S4. Analysis of self-rectifying characteristics and read margin in a crossbar array. (a) I–V curves demonstrating rectifying behavior under different V_set conditions. (b) Schematic of the worst-case read scenario in a crossbar structure. (c) Calculated read margin versus crossbar array size for various V_set values, confirming 10% margin at 194×194.

A device developed in this study exhibits self-rectifying characteristics that effectively address the sneak current issue in high-density crossbar arrays. A depletion region formed at the bottom electrode under negative bias rectifies the tunneling current, significantly reducing unwanted current flow through unselected paths in the array. This intrinsic self-rectification is essential for suppressing sneak currents and improving the overall reliability of the array.

Figure S4a presents I–V characteristics that compare the rectifying behavior at different set voltages (V_SET_). In crossbar architectures, sneak currents can flow not only through the selected cell but also through unselected cells sharing the same row and column, degrading device performance. To evaluate this effect, we analyzed the worst-case read scenario, where the selected cell is in the high-resistance state (HRS), and all other cells are in the low-resistance state (LRS).

The crossbar array was evaluated under various read schemes, and the I–V curve obtained from the adjusted half-bias read method was used to calculate the read margin (ΔV). The results are presented in Figure S4b. Furthermore, the maximum crossbar line length was analyzed by calculating the read margin using the following equations:

${\left( 1 \right) R}_{sneak}=\frac{R_{LRS}^{sneak}}{N-1}+\frac{R_{LRS}^{sneak}}{{(N-1)}^{2}}+\frac{R_{LRS}^{sneak}}{N-1},$

${\left( 2 \right) R}_{LRS, sensing}=\frac{R_{LRS}^{sneak}\times R_{sneak}}{R_{LRS}^{sneak}+R_{sneak}},$

${\left( 3 \right) R}_{HRS, sensing}=\frac{R_{HRS}^{sneak}\times R_{sneak}}{R_{HRS}^{sneak}+R_{sneak}},$

(4) $\frac{\Delta V}{V_{pu}}=\frac{V_{out, HRS}}{V_{pu}}-\frac{V_{out, LRS}}{V_{pu}}=\frac{R_{pu}}{R_{HRS, sensing}+R_{pu}}-\frac{R_{pu}}{R_{LRS, sensing}+R_{pu}}$

Equation (1) defines the total equivalent resistance related to sneak current paths.

Equations (2) and (3) represent the effective resistance values in the LRS and HRS states, respectively.

Equation (4), derived from Kirchhoff’s law, calculates the read margin as a function of the pull-up voltage (V_pu_).

The results for various V_SET_ values are summarized in Figure S4c.

The proposed device achieves a 10% read margin at V_SET_ = 5.6 V, enabling a maximum crossbar array size of 194 × 194. This self-rectifying behavior allows for the realization of larger crossbar arrays, thereby enhancing integration density and scalability in neuromorphic systems.


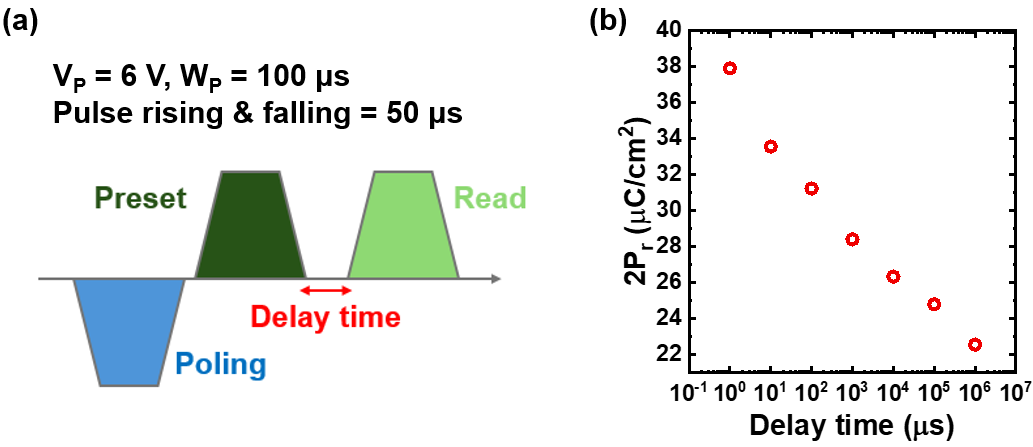


Figure S5. Measurement sequence and retention characteristics of the FM device. a) the pulse scheme used for retention measurements: a prepoling pulse aligns the polarization direction, followed by a preset pulse and a read pulse, with varying delay times introduced between the preset and read pulses. b) the extracted 2P_r_ values as a function of delay time, demonstrating polarization retention loss. At short delay times, the aligned domains remain stable, resulting in higher 2P_r_ values. However, as the delay time increases, depolarization fields cause randomization of aligned domains, leading to a gradual decrease in 2P_r_ values. These results highlight the volatile characteristics of the FM device, advantageous for neuromorphic applications.

**Table S1.** Stretched-exponential fitting parameters for retention loss tests of the fabricated FM device at different V_SET_. The fitting is based on the stretched-exponential model $I(t)=I_{0}exp{(-(t/\tau)}^{\beta}$, where $I_{0}$ represents the initial current, $\tau$ is the relaxation time, and 𝛽 is the stretching exponent. The table summarizes the parameters for V_SET_ values of 5.5 V, 5.7 V, and 6.0 V. Notably, higher V_SET_ values result in a reduced relaxation time 𝜏, indicating faster retention loss, while 𝛽 remains consistent across the conditions.

| Model | Stretched exponential model | | |
| --- | --- | --- | --- |
| Equation | $I(t)=I_{0}exp(-{(t/\tau)}^{\beta}$ | | |
| V_SET_ | $I_{0}$(μA) | $\beta$ | $\tau$ |
| 5.5 V | 3.5 | 0.55 | 2.1 |
| 5.7 V | 5 | 0.55 | 1.3 |
| 6.0 V | 7 | 0.55 | 0.45 |


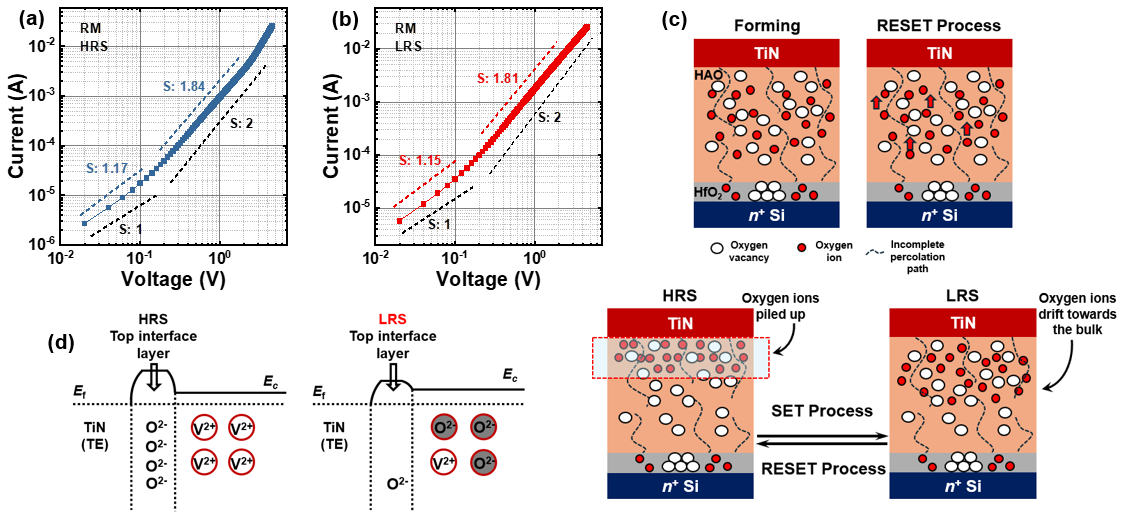


Figure S6. I–V fitting results for the RM device in a) HRS and b) LRS states. The black dotted lines indicate the slopes, and the colored dotted lines represent the linear fitting results in respective regions. c) Schematic diagrams illustrating the resistive switching mechanism, showing the migration of oxygen ions and redox reactions within the HAO layer during forming, SET, and RESET processes. d) Energy band diagrams of the RM device in HRS and LRS states, depicting interfacial barrier changes caused by oxygen ion migration and recombination with oxygen vacancies.

Figures S6a,b show the fitted I‒V plots for the RM device in both high-resistance state (HRS) and low-resistance state (LRS), respectively. The results reveal Ohmic conduction behavior in the low-voltage range (below 0.2 V) and quadratic behavior in the high-voltage range, as observed on a double-logarithmic scale. The transport mechanism in the RM device follows the space-charge-limited current (SCLC) model,^[S1,S2]^ which is characterized by two distinct regimes. In the low-electric field regime, the current is primarily governed by thermally generated free electrons within the dielectric layer. In contrast, at higher electric fields exceeding a critical threshold, the density of injected free electrons from the electrodes surpasses the equilibrium concentration, resulting in the accumulation of excess electrons between the electrodes. When the applied voltage reaches a critical value, this space charge limits the total current flow. Figure S6c provides a schematic representation of the resistive switching mechanism in the RM device, informed by its structural characteristics, I‒V behavior, and fitting results. During the forming process, the thinner HfO_2_ layer undergoes hard breakdown due to its smaller thickness and lower dielectric constant, leading to the formation of an incomplete percolation path within the HAO layer. Under a sufficient electric field, a significant number of oxygen vacancies and oxygen ions are generated. During the RESET process, these oxygen ions migrate towards the anode, accumulating at the upper interface and transitioning the device to the HRS. Conversely, when a negative voltage is applied to the top electrode, the accumulated oxygen ions at the interface migrate into the bulk, where they combine with oxygen vacancies, switching the device to the LRS. Finally, as shown in Figure S6d, resistive switching is driven by changes in the effective barrier height and/or width at the interface, caused by the migration of oxygen ions and the associated redox reactions with oxygen vacancies. This mechanism highlights the critical role of interfacial dynamics in enabling the observed resistive switching behavior.^[S3,S4]^

^
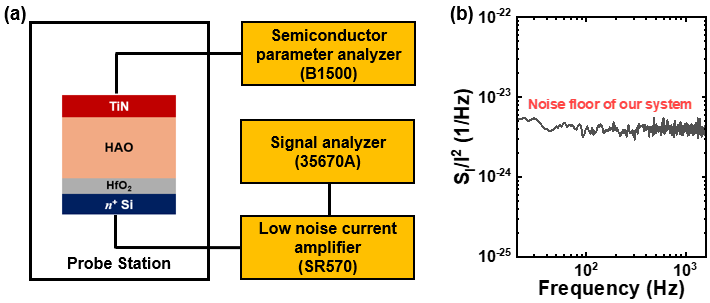
^

Figure S7. a) Schematic diagram of the low-frequency noise (LFN) measurement system. b) Noise floor of the measurement setup.

Figure S7 shows the schematic of the LFN measurement system. The configuration consists of the following major components: 1) Keysight B1500A semiconductor parameter analyzer, 2) Stanford Research SR570 low-noise current amplifier, and 3) an Agilent 35670A dynamic signal analyzer.

The bias applied to the top electrode of DUT was supplied by the B1500A. The device’s low-noise current was amplified by the SR570 and converted into a voltage fluctuation. The 35670A then converted the dynamic signal from the SR570 into a power spectral density by a fast Fourier transform-based spectrum analysis. The noise floor of our measurement system was approximately 10^-24^ A^2^/Hz, which was significantly lower than the device noise. This ensures that the noise power spectral density measured in this study is not affected by the measurement system's noise floor.

^
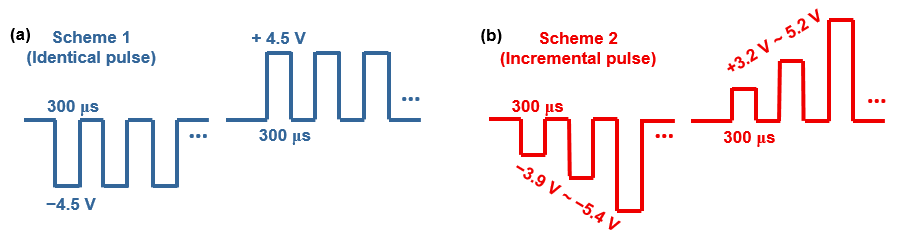
^

Figure S8. Schematic representation of the two pulse schemes: a) Scheme 1 using identical pulses with constant amplitude and b) Scheme 2 using incremental pulses with varying amplitudes.

^
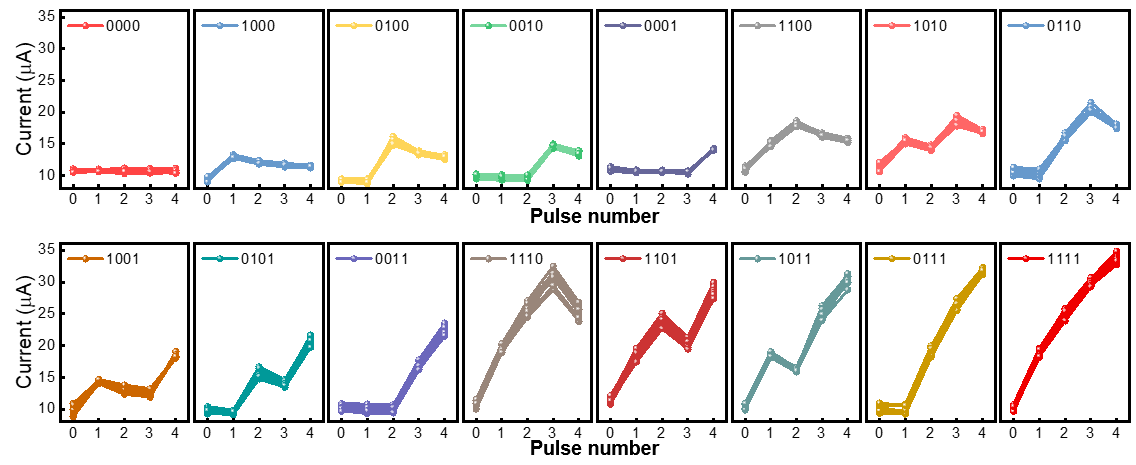
^

Figure S9. Current responses of FM devices to 16 distinct input pulse sequences at each pulse stage (P1–P4), measured over 10 repeated cycles. The results demonstrate successful differentiation of 16 reservoir states with excellent C2C variability of up to 3.52%, confirming the uniformity and reproducibility of the reservoir states.

^
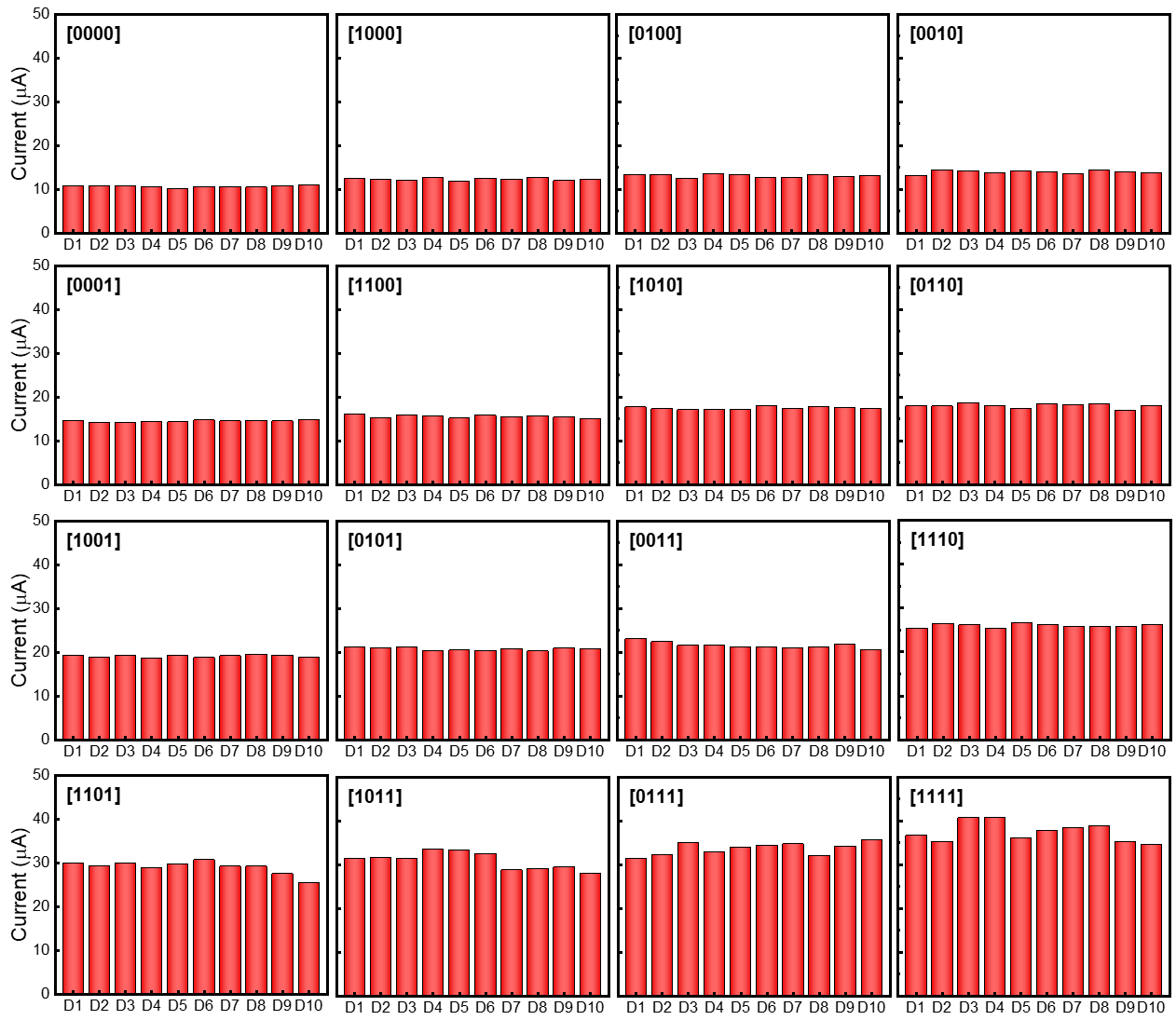
^

Figure S10. Variations across 10 randomly selected FM devices for 16 distinct input pulse sequences. The current responses of the devices were recorded, demonstrating consistent and reproducible behavior across different devices. The results confirm the uniformity of device performance, showcasing minimal variability between devices under identical conditions.

^
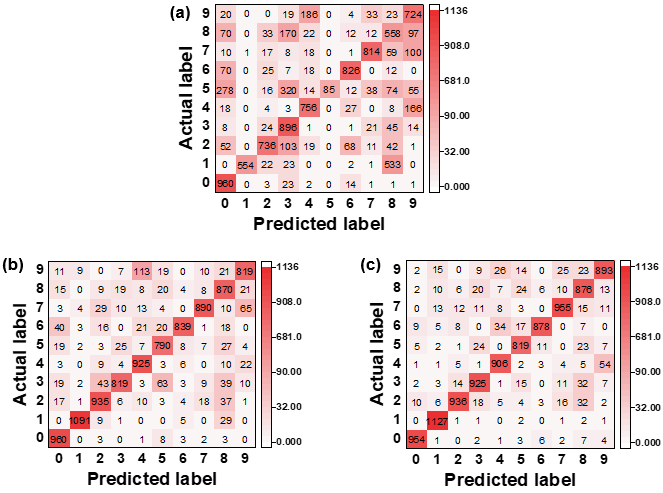
^

Figure S11. Representative confusion matrices for handwritten digit classification under various readout network configurations. a) Results using the identical pulse scheme, showing suboptimal classification accuracy (53.7%) due to limited linearity in weight updates. b) Results with the incremental pulse scheme, demonstrating significant improvement in classification accuracy (88.2%) through enhanced linearity and optimized weight updates. c) Results from offline training using ISPVA with 50 conductance states, achieving superior classification accuracy of 93.3%, comparable to software-based systems (95%).

Supplementary Note 1: Nonlinearity factor calculation

Wang et al. proposed the following Nonlinearity (NL) factor definition, which calculates the maximum deviation between normalized potentiation and depression conductance values:^[S5]^

$$\boldsymbol{NL}\mathbf{=}\boldsymbol{Max}\left| \boldsymbol{G}_{\boldsymbol{P}}\mathbf{(}\boldsymbol{n}\mathbf{)-}\boldsymbol{G}_{\boldsymbol{D}}\mathbf{(}\boldsymbol{n}\mathbf{)} \right|\mathbf{,}\boldsymbol{n}\boldsymbol{=1, 2, 3, \cdot\cdot\cdot,}\boldsymbol{N}$$

where $\boldsymbol{G}_{\boldsymbol{P}}\boldsymbol{(n)}$ and $\boldsymbol{G}_{\boldsymbol{D}}\boldsymbol{(n)}$ represent the normalized conductance values during potentiation and depression processes, respectively. $\boldsymbol{N}$ denotes the total number of pulses. By definition, an NL value of zero indicates perfect linearity, where the conductance updates align precisely with an ideal linear model. However, this definition does not differentiate the distinct patterns of potentiation and depression. For example, if $\boldsymbol{G}_{\boldsymbol{P}}\boldsymbol{(n)}$ is concave with a positive slope and $\boldsymbol{G}_{\boldsymbol{D}}\boldsymbol{(n)}$ is concave with a negative slope, the NL factor may fail to accurately represent their individual nonlinearities.

To address these limitations, we introduce a modified NL factor that separates the evaluation of potentiation and depression. The modified NL factors,$\boldsymbol{\alpha}_{\boldsymbol{p}}$ and $\boldsymbol{\alpha}_{\boldsymbol{d}}$, are defined as:

For potentiation (LTP):

$$\boldsymbol{\alpha}_{\boldsymbol{p}}\mathbf{=}\boldsymbol{Max}\left| \boldsymbol{G}_{\boldsymbol{P}}\mathbf{(}\boldsymbol{n}\mathbf{)-(}\frac{\boldsymbol{n}}{\boldsymbol{N}}\boldsymbol{)} \right|\mathbf{,}\boldsymbol{n}\boldsymbol{=1, 2, 3, \cdot\cdot\cdot,}\boldsymbol{N}$$

For depression (LTD):

$$\boldsymbol{\alpha}_{\boldsymbol{d}}\mathbf{=}\boldsymbol{Max}\left| \boldsymbol{G}_{\boldsymbol{D}}\mathbf{(}\boldsymbol{n}\mathbf{)-(1-(}\frac{\boldsymbol{n}}{\boldsymbol{N}}\boldsymbol{)} \right|\mathbf{,}\boldsymbol{n}\boldsymbol{=1, 2, 3, \cdot\cdot\cdot,}\boldsymbol{N}$$

where $\mathbf{(}\frac{\boldsymbol{n}}{\boldsymbol{N}}\boldsymbol{)}$ is $\mathbf{1-(}\frac{\boldsymbol{n}}{\boldsymbol{N}}\mathbf{)}$ the ideal linear conductance model for potentiation and depression, respectively, representing evenly distributed conductance changes across the total number of pulses ($\boldsymbol{N}$).

This modified approach evaluates the maximum deviation of conductance values from the ideal linear model separately for LTP and LTD processes, allowing for a more precise characterization of nonlinear behavior.

Supplementary Note 2: Physical origins of volatility in the ferroelectric memristor device

The volatility observed in our ferroelectric memristor (FM) device, despite employing a heavily doped n^+^ Si bottom electrode and a high-k interlayer, can be attributed to the following combined physical mechanisms.

1. Material Defects and Interfacial Depolarization

The HfAlO (HAO) switching layer is prone to material defects such as oxygen vacancies and incomplete crystallization, which induce local internal fields that disturb polarization alignment.

At the HAO/n^+^ Si interface, these defects hinder perfect charge screening, resulting in the formation of interfacial depolarization fields that accelerate spontaneous polarization relaxation.^[S6,S7]^ Material defects are identified as the dominant origin of the observed volatility.

2. Charge Trapping Effects

During device operation, mobile carriers can become trapped at bulk or interface defect sites within the HAO layer. The resulting localized space charges distort the internal electric field and destabilize the ferroelectric polarization. Under repeated pulsed operation, characteristic of reservoir computing, dynamic charge trapping and detrapping significantly enhance the apparent volatility.^[S8,S9]^

3. Limited Polarization Stabilization in the HAO Layer

The ferroelectric phase in ultrathin HAO films tends to be metastable at room temperature. Small electrical or mechanical perturbations can easily induce back-switching or domain relaxation.^[S10]^ Additionally, grain boundaries and local non-uniformities arising from the ALD process may further promote polarization collapse.

4. Transient Capacitive Charging and Leakage Currents

Fast pulse measurements emphasize transient capacitive charging and leakage currents. These transient effects can cause a rapid decay of the measured current, mimicking fast polarization relaxation even if intrinsic polarization partially remains.^[S11,S12]^

Supplementary References

[S1] C. P. Kwan, M. Street, A. Mahmood, W. Echtenkamp, M. Randle, K. He, J. Nathawat, N. Arabchigavkani, B. Barut, S. Yin, R. Dixit, U. Singisetti, C. Binek, J. P. Bird, AIP Advances 2019, 9, 055018.

[S2] P. Zhang, Y. S. Ang, A. L. Garner, Á. Valfells, J. W. Luginsland, L. K. Ang, Journal of Applied Physics 2021, 129, 100902.

[S3] H. Y. Jeong, J. Y. Lee, S.-Y. Choi, J. W. Kim, Appl. Phys. Lett, 2009, 95,162108.

[S4] X. L. Shao, L. W. Zhou, K. J. Yoon, H. Jiang, J. S. Zhao, K. L. Zhang, S. Yoo, C. S. Hwang, Nanoscale, 2015, 7, 11063.

[S5] I-T. Wang, C.-C. Chang, L.-W. Chiu, T. Chou, T.-H. Hou, Nanotechnology, 2016, 27, 365204.

[S6] J. Lee, K. Yang, J. Y. Kwon, J. E. Kim, D. I. Han, D. H. Lee, J. H. Yoon, M. H. Park, Nano Convergence 2023, 10, 55.

[S7] F. Yan, Y. Wu, Y. Liu, P. Ai, S. Liu, S. Deng, K.-H. Xue, Q. Fuad, W. Dong, Mater. Horiz. 2024, 11, 626.

[S8] M. Pesic, S. Slesazeck, T. Schenk, U. Schroeder, T. Mikolajick, Phys. Status Solidi A 2016, 213, 2, 270.

[S9] E. Yurchuk, J. Müller, S. Müller, J. Paul, M. Peši´c, R. V. Bentum, U. Schroeder, T. Mikolajick, IEEE Transactions on Electron Devices 2016, 63, 9, 3501.

[S10] T.-Y. Wang, W.-C. Kao, Y.-T. Yin, J. Shieh, M.-J. Chen, Journal of the European Ceramic Society 2022, 42, 4221.

[S11] K. D. Kim, Y. J. Kim, M. H. Park, H. W. Park, Y. J. Kwon, Y. B. Lee, H. J. Kim, T. Moon, Y. H. Lee, S. D. Hyun, B. S. Kim, C. S. Hwang, Adv. Funct. Mater. 2019, 29, 1808228

[S12] P. Sharma, J. Zhang, A. K. Saha, S. Gupta and S. Datta, 2017 75th Annual Device Research Conference (DRC), South Bend, IN, USA, 2017, pp. 1-2.
